# Supplementary material for: Selective control of synaptic plasticity in heterogeneous networks through transcranial alternating current stimulation (tACS)
Source: PLoS Comput Biol. 2023 Apr 27;19(4):e1010736. doi: 10.1371/journal.pcbi.1010736 (PMC10168590; doi:10.1371/journal.pcbi.1010736)
Supplement: Attachment — Submitted filename: Reply to comments.pdf [file pcbi.1010736.s003.pdf]

AUTHORS REPLY TO THE REVIEWERS

Aref Pariz[1,3], Daniel Trotter[2,3], Axel Hutt[4], Jeremie lefebvre[1,2,3,5]

March 13, 2023

[1] Department of Biology, University of Ottawa, ON, Canada [2]Department of Physics, University of Ottawa, ON, Canada [3]Krembil Brain Institute, University Health Network, Toronto, ON, Canada [4]Team MIMESIS, INRIA, UMR7357 CNRS, ICube Research Department, F-67085 Strasbourg, France [5]Department of Mathematics, University of Toronto, ON, Canada

Dear Editors,

We express our gratitude to the editors and reviewers for the time and effort in handling and reviewing our manuscript. We worked diligently to address all concerns raised, and we provide a point by point response to those below. We express our gratitude to the editors and reviewers for the time and effort in handling and reviewing our manuscript. We worked diligently to address all concerns raised, and we provide a point by point response to those below.

Kind regards,

Aref Pariz, on behalf of the authors

Editor's Comment

Comment

Please consider using a different colormap in figure 3, one which is sequential and perceptually uniform.

Answer

Done. The Fig. 3(B) has been updated with an uniform colormap.

1 Reviewer 1

1.1 Comment

This manuscript describes a really quite elegant series of simulations investigating the effect of timescale heterogeneity on synaptic plasticity during tACS. The simulations are based on Leaky-Integrate-and-Fire (LIF) excitatory and inhibitory model neurons to develop two neuron network motifs, intra- and inter-laminar cortical circuit models. Neurons and/or networks are assumed to be in asynchronous spiking state to quantify tACS-induced synaptic changes in the absence of endogenous oscillations. Plasticity in the network is modelled using Hebbian spike-timing dependent plasticity (STDP). The results demonstrate that time scale variations through membrane time constant heterogeneity enables tACS to guide inter-neuronal, intra-laminar and inter-laminar synaptic plasticity in a directional and frequency-specific manner. Stimulation using tACS leads to entrainment and the consequent directional synaptic weight modification such that the synapses between average and fast neutrons undergo LTD, while those between average and slow neutrons undergo LTP, irrespective of cell type. The magnitude and direction of overall synaptic weights is stimulation frequency-dependent. Their model has certain limitations, including but not limited to simplified cortical connectivity, unaccounted cell-specific variations and weak connectivity regime. Nonetheless, this interdisciplinary framework makes a strong case for the timescale heterogeneity as a means of guiding synaptic plasticity using non-invasive stimulation paradigm (tACS).

Answer

We would like to thank the Reviewer for their insightful, constructive and positive criticism on our work. We believe addressing this Reviewer's concerns has improved the manuscript's results, quality and scope. We addressed each of the reviewer's comments directly below.

1.2 Comment

Computational code used for this study and their data are not publicly available. Please ensure it is available without any restriction.

Answer

We apologize for the oversight. The codes are now fully available publicly on GitHub `https://github.com/arefpz/neuronal_population`. This link has also been added to our revised manuscript.

1.3 Comment

Results- page 3- line 92 - Please consider expanding the sentence rather than putting the second condition in brackets, to ensure a smooth flow. Neurons

with smaller (larger)....shorter (longer)... Consider replacing it with ‘neurons with smaller MTC exhibit shorter integration times and those with larger MTC exhibit longer integration times’, or other format that you might like.

Answer

We apologize for the confusion. The text has been corrected (see lines 96-97).

1.4 Comment

Fig 3 legend: The top (bottom)... ‘The top and bottom circles denote the MTC of neuron(2) and neuron (1), respectively.’

Answer

The text has been modified (see caption on page 7).

1.5 Comment

Please stay consistent. At some places, Fig X (Y#) is used, while at other instances, it is Fig X Y#

Answer

The text has been modified.

1.6 Comment

Discussion: Page 10, line 338 - Please modify the sentence: ‘as well as a other. . . , ought to play’.

Answer

Thank you. The text has been corrected (”ought” changed to ”ought to”; see lines 389).

2 Reviewer 2

2.1 Comment

Pariz et al. build heterogeneous neural network models to explore the effects of transcranial alternating current stimulation (tACS) on neural behavior. This is an interesting and sufficiently novel research goal, however, there are several principal weaknesses. The network models are not clearly described, I do see any constrains to their parameters, and they demonstrate behavior that is known to not exist in the real neural networks during tACS. Thus, interpretability of the results is not high.

Answer

We would like to thank the Reviewer for their insightful and constructive criticism on our work. We believe addressing the Reviewer's concerns has improved the manuscript's results, quality and scope. We addressed each of the reviewer's comments directly below.

2.2 Comment

First of all, I am concerned about the properties of neural networks. From Figure 4E it is evident that the spiking rate increased manifolds during stimulation. That is contrary to the expected behavior - all experimental neural studies (Johnson et al., Science Adv, 2019; Krause et al., PNAS, 2019), as well as computational models (Tran et al., Neuroimage, 2022; Obermayer and colleagues in PLOS Comp. Biol.), showed a little-to-no increase in spiking rate during tACS. Thus, the present models do not capture the critical features of their biological counterparts.

Answer

We appreciate this excellent comment, which lead to additional analyses and simulations from the authors. Our analyses show that the studies cited by the Reviewer and results we report in our study can be reconciled by accounting for differences in (effective) stimulation amplitude.

We have deliberately conducted our experiments in a regime of high (effective) tACS amplitude to expose neural populations to currents sufficient to cause depolarization. This has been done to amplify resulting synaptic changes resulting from STDP to characterize how heterogeneity and tACS interact at temporal scales accessible by our simulations. Our goal was indeed to quantify how plasticity depends on both cellular MTC heterogeneity and stimulation parameters (e.g., amplitude, frequency). By construction, our model is devoid of many anatomical/physical/experimental constraints that are known to hinder tACS efficacy and overall signal-to-noise ratio on membrane potential, such as skull shunting [1, 2] and cellular orientation [3, 4, 5] - which certainly influences the (effective) magnitude of neural responses to tACS. We now state this explicitly in our revised manuscript to avoid any confusion (see lines 231-236, 330-367, 519-546).

To address the Reviewer's comment, new rather extensive simulations were performed by the authors across a range of smaller stimulation amplitudes to examine the relationship between stimulation amplitude, induced changes in firing rates and synaptic modifications(if any). These analyses collectively show that stimulation-induced synaptic modifications expectedly scale with tACS amplitudes - contextualizing our results with those cited above [3, 6, 7, 8]. Our analyses confirmed that phase alignment (as reported by [3, 6, 7, 8]) represents a generic feature of stimulated neural populations' response in periodic forcing, that is certainly captured by our network model(s) as well. For weak

tACS stimulation amplitudes, no (significant) increase in firing rate could be observed in our simulations, despite noticeable locking between network response and stimulation phases - fully in line with observations reported in [3]. Small yet noticeable frequency- and MTC-specific changes in synaptic coupling could nonetheless be observed (see S1 and S2 in our revised manuscript). Decreasing tACS amplitude further suppressed changes in synaptic weights, while preserving phase locking. At such low tACS amplitudes, entrainment can still be observed, but the loss of cellular specificity in spike timing does not yield significant synaptic modification.

These analyses and conclusions point to the fact that, while it is certainly possible to entrain neural population at small tACS amplitudes without altering their net firing rate, our simulations suggest that such approaches may fail to modify synaptic weights, at least from the perspective of STDP and MTCs heterogeneity.

The point raised by the reviewer, as well new analyses motivated by it, warrant a thorough discussion in our revised manuscript. As such, we now 1) provide a clear context for our simulations in both the Introduction and Results sections (lines 67, 141-143, 195-197, 231-236); 2) added the results of the above analyses in a supplementary material section and refer to it in the Results section (lines 518-546, Figs. S1 and S2); and 3) thoroughly discuss this as well as associated limitations in the Discussion (lines 330-367).

2.3 Comment

The description of the computational network models is confusing. Figure 1 depicts realistic neurons, and some parts of the paper give the impression that two networks of realistic neurons were simulated. However, other methods described radically more simple leaky integrate-and-fire (LIF) neurons. The authors should avoid misrepresentation.

Answer

We apologize for the confusion. We modified Fig. 1 and removed the representation of cortical layers to avoid any misunderstanding. We emphasize in the Abstract, Introduction, Fig 1 caption (and associated discussion in the text of our revised manuscript; see lines 73, 101, and 414) that our analysis relies on LIF neuron models, avoiding any further ambiguity. Variability in the neurons MTC across cortical layers being a central point of our work, we kept panel A of Fig. 1 which contains empirical evidence of this variability.

2.4 Comment

Finally, the authors provided no constraints for the computational parameters in their network models.

Answer

We apologize for the confusion and have clarified this in our revised manuscript. Our model is informed by well established parameter sets gathered from the both the experimental [9, 10, 11, 12] and modelling (i.e., LIF) [13, 14, 15, 16] literature on cortical networks. In addition, to stabilize the network in a balanced state with Asynchronous Irregular[17] activity with weak coupling, we determined the associated (baseline) synaptic weights numerically, ensuring that network stability is maintained and that asymmetrical synaptic weights changes would nonetheless preserve balance. Resulting values are within those reported in other LIF network studies[13]. We kept the coupling week to avoid unbalancing synaptic current due to asymmetrical synaptic weight modifications. We have fully revised our materials and method section, emphasizing the rationale behind the choice of all parameters as well as how these constraints were handled, providing both the relevant reference and more thorough explanation about how they were selected. We lastly note that all parameters are summarized in Table 2.

References

[1] Liu A, Vöröslakos M, Kronberg G, Henin S, Krause MR, Huang Y, et al. Immediate neurophysiological effects of transcranial electrical stimulation. Nature communications. 2018;9(1):5092.

[2] Fernandez-Corazza M, Turovets S, Muravchik C. Spatial sensitivity of the optimal dose in transcranial electrical stimulation to the skull and scalp conductivity specifications. Brain Stimulation: Basic, Translational, and Clinical Research in Neuromodulation. 2019;12(2):581.

[3] Krause MR, Vieira PG, Csorba BA, Pilly PK, Pack CC. Transcranial alternating current stimulation entrains single-neuron activity in the primate brain. Proceedings of the National Academy of Sciences. 2019;116(12):5747–5755.

[4] Radman T, Ramos RL, Brumberg JC, Bikson M. Role of cortical cell type and morphology in subthreshold and suprathreshold uniform electric field stimulation in vitro. Brain stimulation. 2009;2(4):215–228.

[5] Terzuolo C, Bullock T. Measurement of imposed voltage gradient adequate to modulate neuronal firing. Proceedings of the national Academy of Sciences. 1956;42(9):687–694.

[6] Tran H, Shirinpour S, Opitz A. Effects of transcranial alternating current stimulation on spiking activity in computational models of single neocortical neurons. Neuroimage. 2022;250:118953.

[7] Johnson L, Alekseichuk I, Krieg J, Doyle A, Yu Y, Vitek J, et al. Dose-dependent effects of transcranial alternating current stimulation on spike timing in awake nonhuman primates. Science advances. 2020;6(36):2747.

[8] Cakan C, Obermayer K. Biophysically grounded mean-field models of neural populations under electrical stimulation. PLoS computational biology. 2020;16(4):e1007822.

[9] Thomson AM, Bannister AP. Interlaminar connections in the neocortex. Cerebral cortex. 2003;13(1):5–14.

[10] Campagnola L, Seeman SC, Chartrand T, Kim L, Hoggarth A, Gamlin C, et al. Local connectivity and synaptic dynamics in mouse and human neocortex. Science. 2022;375(6585):eabj5861.

[11] Moradi Chameh H, Rich S, Wang L, Chen FD, Zhang L, Carlen PL, et al. Diversity amongst human cortical pyramidal neurons revealed via their sag currents and frequency preferences. Nature communications. 2021;12(1):1–15.

[12] Institute A. Dataset: Allen Institute for Brain Science. Allen Cell Types Database – Human Morphology-Electrophysiology. 2015;.

[13] Brunel N, Wang XJ. What determines the frequency of fast network oscillations with irregular neural discharges? I. Synaptic dynamics and excitation-inhibition balance. Journal of neurophysiology. 2003;90(1):415–430.

[14] Tuckwell HC. Cortical network modeling: Analytical methods for firing rates and some properties of networks of LIF neurons. Journal of Physiology-Paris. 2006;100(1-3):88–99.

[15] Burkitt AN. A review of the integrate-and-fire neuron model: I. Homogeneous synaptic input. Biological cybernetics. 2006;95:1–19.

[16] Gerstner W, Kistler WM, Naud R, Paninski L. Neuronal dynamics: From single neurons to networks and models of cognition. Cambridge University Press; 2014.

[17] Renart A, De La Rocha J, Bartho P, Hollender L, Parga N, Reyes A, et al. The asynchronous state in cortical circuits. science. 2010;327(5965):587–590.
